# iTRAQ-Based Comparative Proteomic Analysis Provides Insights into Molecular Mechanisms of Salt Tolerance in Sugar Beet (*Beta vulgaris* L.)

**DOI:** 10.3390/ijms19123866

**Published:** 2018-12-04

**Authors:** Guo-Qiang Wu, Jin-Long Wang, Rui-Jun Feng, Shan-Jia Li, Chun-Mei Wang

**Affiliations:** 1 School of Life Science and Engineering, Lanzhou University of Technology, Lanzhou 730050, China; jinlongwang0112@163.com (J.-L.W.); fengrj143202@163.com (R.-J.F.); lsjlut@163.com (S.-J.L.); 2 Lanzhou Institute of Husbandry and Pharmaceutical Science, CAAS, Lanzhou 730050, China; wangchunmeilzmys@163.com

**Keywords:** sugar beet, proteomics, salt tolerance, relative labeling and absolute quantification of isotope (iTRAQ)

## Abstract

Salinity is one of the major abiotic stress factors that limit plant growth and crop yield worldwide. To understand the molecular mechanisms and screen the key proteins in response of sugar beet (*Beta vulgaris* L.) to salt, in the present study, the proteomics of roots and shoots in three-week-old sugar beet plants exposed to 50 mM NaCl for 72 h was investigated by isobaric Tags for Relative and Absolute Quantitation (iTRAQ) technology. The results showed that 105 and 30 differentially expressed proteins (DEPs) were identified in roots and shoots of salt-treated plants compared with untreated plants, respectively. There were 46 proteins up-regulated and 59 proteins down-regulated in roots; and 13 up-regulated proteins and 17 down-regulated proteins found in shoots, respectively. These DEPs were mainly involved in carbohydrate metabolism, energy metabolism, lipid metabolism, biosynthesis of secondary metabolites, transcription, translation, protein folding, sorting, and degradation as well as transport. It is worth emphasizing that some novel salt-responsive proteins were identified, such as PFK5, MDH, KAT2, ACAD10, CYP51, F3H, TAL, SRPR, ZOG, V-H^+^-ATPase, V-H^+^-PPase, PIPs, TIPs, and tubulin α-2/β-1 chain. qRT-PCR analysis showed that six of the selected proteins, including BvPIP1-4, BvVP and BvVAP in root and BvTAL, BvURO-D1, and BvZOG in shoot, displayed good correlation between the expression levels of protein and mRNA. These novel proteins provide a good starting point for further research into their functions using genetic or other approaches. These findings should significantly improve the understanding of the molecular mechanisms involved in salt tolerance of sugar beet plants.

## 1. Introduction

With the intensification of climate change, soil salinization has become a global problem. Excessive salt can cause damage to most plants, especially glycophytes, which is mainly caused by its osmotic potential, ion poisoning, and secondary stress on plants [1]. Increasing salt leads to a decrease in biomass and a serious reduction in crop yields [2]. Plants have developed a series of mechanisms in response to salt stress. It is documented that plants can accumulate soluble solute molecules, such as sugars, amino acids and/or other compounds, in order to protect themselves from salt stress. Plants, especially some halophytes, also can accumulate more ions (Na^+^, K^+^, and Ca^2+^) to balance osmotic stress induced by salt stress [3]. Therefore, exploring the mechanisms of salt tolerance in plants is of great significance for improving salt tolerance of species and cultivating salt-tolerant varieties.

To date, many studies have been done to elucidate the mechanisms of salt tolerance in plants. However, there is still a lot of work that needs to be further refined to determine the key factors of how plants respond to salt stress. When exposed to salt, plants produce a series of changes in the transcription, metabolic pathways, and related genes and proteins. At the cellular and molecular levels, salt-responsive proteins in different plants have been identified using proteomic methods. These proteins included those involved in transcription and regulation factors, osmoregulatory factors, hormones, and oxidative stress-related proteins, chaperones, and transporters [4].

High-throughput sequencing technology is a powerful tool for the identification of salt-tolerant proteins, the study of complex physiological processes, and the elucidation of salt-tolerant mechanisms. Proteomic analysis is usually performed by protein separation and visualization of proteins and mass spectrometry (MS) combined with two-dimensional gel electrophoresis (2-DE). Although this technique has strong, mature, and sensitive features, there are still questions about its ability to identify all elements of the proteome [5]. The isobaric Tags for Relative and Absolute Quantitation (iTRAQ) technology is a new proteomics methodology that enables up to eight samples to be quantified in a single experiment. This quantitative method, with high quantitative accuracy features, can quantify virtually any protein sample. Since its first presentation at the American Mass Spectrometry Annual Conference in 2004, it has become an increasingly and widely used quantitative proteomics technology [6,7,8,9].

Sugar beet (*Beta vulgaris* L.) is one of the major sugar crops that supplies 30% of the world’s annual sugar production and is an important source of fuel ethanol and animal feed [10,11,12,13]. This species, which belongs to the order of Caryophylalles, is diploid with 2*n* = 18 chromosomes, has a genome size of 714–758 Mb, and its whole genome sequencing was completed in 2014 [14]. It is well known that sugar beet is very sensitive to salt at its seedling stage [15,16]. The analysis of protein changes in sugar beet subjected to salt stress has important implications for further elucidation and improvement of its salt tolerance. Although the proteomics analysis of sugar beet under salt stress has been reported, most of the researchers mainly relied on 2-DE for protein separation [17,18,19,20]. Li et al. [21] first analyzed the changes of membrane proteins using the iTRAQ method in sugar beet monosomic addition line M14. However, there have been still insufficient reports on the application of iTRAQ technology to study sugar beet response to salt stress. Wu et al. [22,23] evaluated salt tolerance of three cultivars in sugar beet using proline, soluble sugars, and cation accumulation criteria, and found that cultivar ‘Gantang7’ was more tolerant to salt- and drought-stresses than other cultivars. Interestingly, it was observed that addition of 50 mM NaCl can stimulate the growth and enhance resistance to osmotic stress induced by sorbitol in ‘Gantang7’ [24]. However, the molecular regulation mechanisms of salt response in sugar beet are still far from being understood.

In the present study, iTRAQ-based quantitative proteomic analysis was employed to identify the proteins that are involved in salt response in sugar beet cultivar ‘Gantang7’. Differentially expressed proteins (DEPs) in roots and shoots at 72 h after 50 mM NaCl treatment and untreated controls were identified and quantified. To elucidate the function of the DEPs in the salt-response process, the functions and pathways of identified DEPs were analyzed by Gene Ontology (GO) and Kyoto Encyclopedia of Genes and Genomes (KEGG) methods. The results of the study should provide important insights into the underlying molecular mechanisms of salt tolerance in sugar beet.

## 2. Results

### 2.1. Protein Identification Information by iTRAQ

iTRAQ technology was used to analyze and compare the differentially expressed proteins in roots and shoots of salt-treated sugar beet plants and untreated plants. A total of 266,862 and 249,800 spectra were generated from roots and shoots of sugar beet plants, respectively. In roots, the numbers of matching spectra are 71,299. Of these spectra, 59,676 were identified to be unique spectra. Here, 17,710 peptides were identified with 16,129 unique peptides and 3922 proteins (Figure S1). In shoots, the numbers of matching spectra are 41,489. Among these, 35,515 were found to be unique peptides while 9136 peptides were identified with 8366 unique peptides and 2510 proteins (Figure S1). These results suggested that the iTRAQ has high degree of sensitivity, it could obtain more comprehensive information than other techniques when used to analyze the proteins in sugar beet.

The statistical analyses of all the identified proteins in roots and shoots of sugar beet were conducted according to the relative molecular weights (Figure S2). The mass of the identified proteins showed a normal distribution, with 10–50 kDa, 50–100 kDa, and 100 kDa proteins accounting for 53%, 35%, and 12% in roots, and 57%, 34%, and 9% in shoots, respectively (Figure S2). These results indicated that the proteins’ molecular weight distributions, which were identified by iTRAQ, were relatively broad, and covered the sizes of the different proteins. The peptide number distribution of proteins (number of amino acid) with 3–5 peptides, 6–10 peptides, and 11 or more peptides comprised 36, 4456, and 12,361 in roots, and 58, 3194, and 5484 in shoots, respectively, most peptides have 8–15 amino acids, and few peptides have five or less amino acids (Figure S3).

The distribution of protein sequence coverage was analyzed for all of the proteins identified by iTRAQ. The results showed that the distribution of protein sequence coverage with 40–100%, 30%– 40%, 20–30%, 10–20%, and under 10% variations accounted for 8.0%, 8.0%, 13.2%, 23.9%, and 46.9% in roots (Figure S4a), and 4.0%, 6.1%, 12.4%, 22.9%, and 54.5% in shoots (Figure S4b), respectively. These results suggested that iTRAQ was able to cover the majority of the expressed proteins. The peptide number analysis for the proteins identified by iTRAQ showed that the peptide number distribution of the proteins with 1–5 peptides, 6–10 peptides, and 11 or more peptides comprised 2915, 591, and 416 in roots, and 2041, 323, and 146 in shoots, respectively (Figure S5). It was also observed that the number of proteins decreased with the increased number of matched peptides (Figure S5). Of these peptides, more than 48.2% (1890/3920) and 53.5% (1343/2510) of the proteins in roots and shoots, respectively, contained at least two peptides (Figure S5). These results suggested that the proteins’ isolation and identification were satisfactory.

### 2.2. Identification of Differentially Expressed Proteins (DEPs)

**Figure 1:**
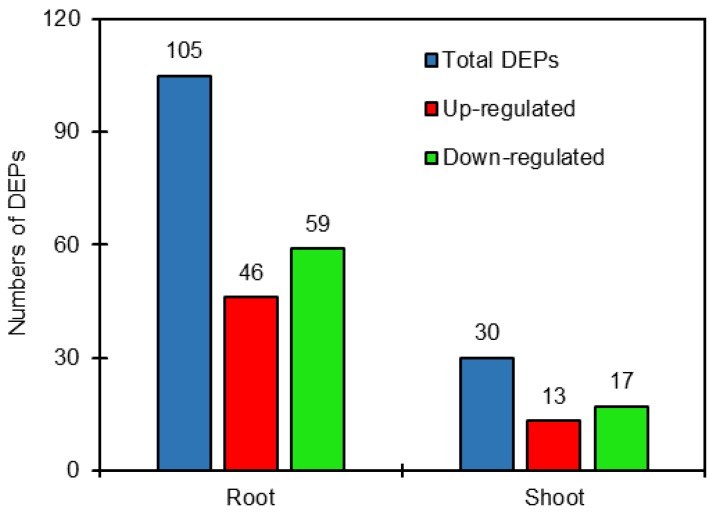
Number of differentially expressed proteins (DEPs) in roots and shoots of salt-treated sugar beet plants compared with untreated plants.

DEPs were defined as those with a >1.2-fold or <0.8-fold changes in relative abundance (*p* < 0.05) between salt-treated plants and control plants in sugar beet. The results showed that the 105 DEPs were identified comparing salt treatment with the control in roots, of which 46 proteins were up-regulated and 59 proteins were down-regulated. Also, 30 DEPs were identified in shoots comparing salt treatment with the control, including 13 up-regulated and 17 down-regulated proteins (Tables S1 and S2, Figure 1). It is clear that the DEPs in roots were obviously more than those in shoots.

**Figure 2:**
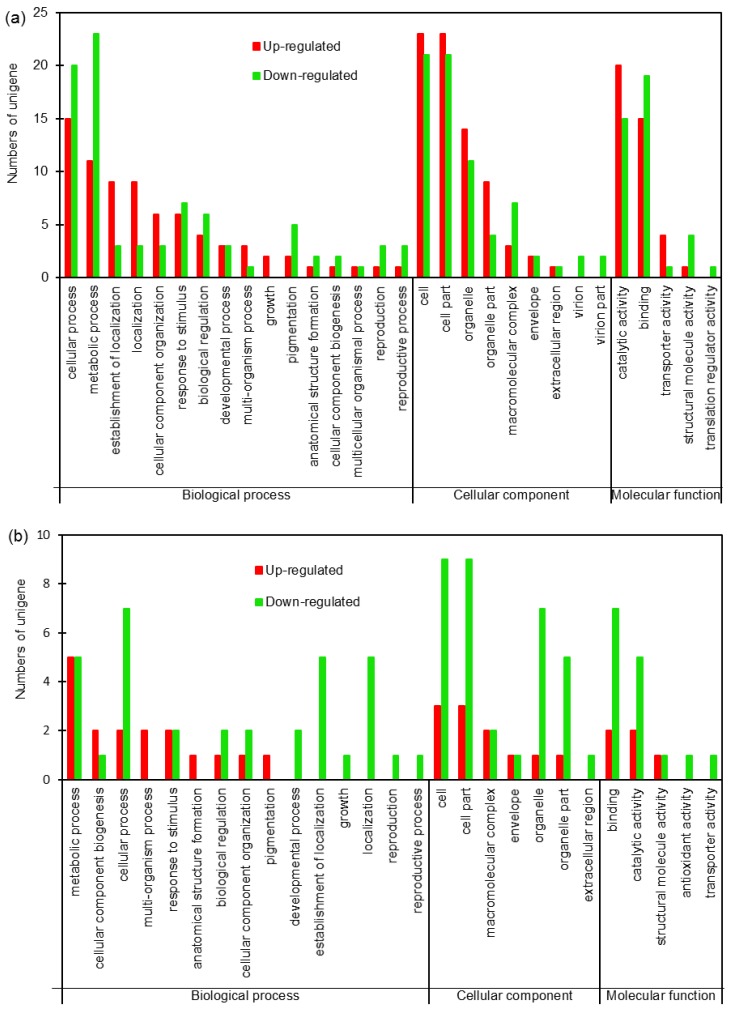
Gene Ontology (GO) analysis of the DEPs in roots (**a**) and shoots (**b**) of sugar beet exposed to salt condition. Red and green bars represent up- and down-regulated.

To further investigate the biological processes, subcellular localization and molecular functions of the DEPs, Gene Ontology (GO) analysis was performed on the 105 and 30 DEPs in roots and shoots of sugar beet using Blast2go software (version 2.5). The results showed that DEPs in roots and shoots were annotated into 30 and 27 functional groups, respectively, including 16 and 15 biological processes, 9 and 7 cellular components, and 5 and 5 molecular functions respectively (Figure 2a,b). At the biological processes level, the DEPs both in roots and shoots were mainly involved in cellular, metabolic, cellular component organization, response to stimulus, developmental process, and so on. At the cellular component level, the DEPs were mainly focused on the cells, cell part, macromolecular complex, organelle, and so on. At the molecular function level, the DEPs were mainly involved in catalytic activity, binding, transporter activity, structural molecular activity, and so on. These results indicated that salt-responsive proteins were mainly involved in cellular, metabolic processes, response to abiotic, and so on (Figure 2a,b).

### 2.3. Cluster Analysis of DEPs

**Figure 3:**
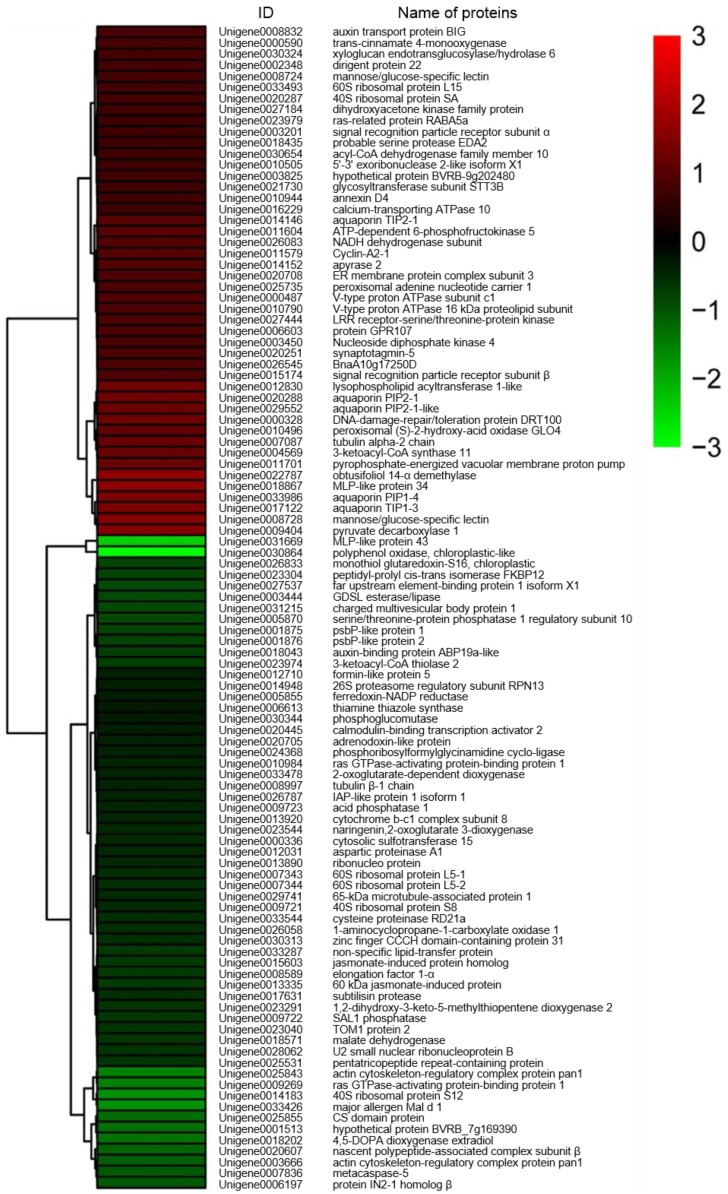
Heat map diagram of expression patterns for differentially expressed proteins in roots of salt-treated sugar beet plants compared with untreated plants. The color scale bar in the right, bottom corner indicates increased (red) and decreased (green) levels in response to salt change.

**Figure 4:**
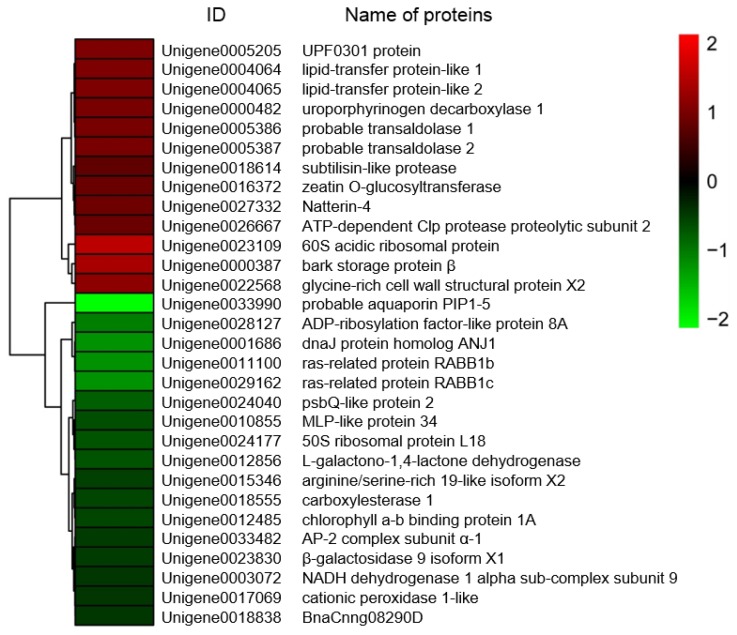
Heat map diagram of expression patterns for differentially expressed proteins in shoots of salt-treated sugar beet plants compared with untreated plants. The color scale bar in the right, bottom corner indicates increased (red) and decreased (green) levels in response to salt change.

Based on fold-changes in protein expression levels, cluster analysis was performed for proteins differentially expressed in roots and shoots of sugar beet using R software (version 3.4.4) (https://www.r-project.org/) (accessed on 2 July 2018). The results showed that expression patterns of DEPs between salt-treated plants and untreated plants were clustered in two modules, down- to increased and up- to down-regulated, both in roots and shoots of sugar beet (Figure 3 and Figure 4).

### 2.4. Kyoto Encyclopedia of Genes and Genomes (KEGG) Enrichment Analysis of DEPs

**Figure 5:**
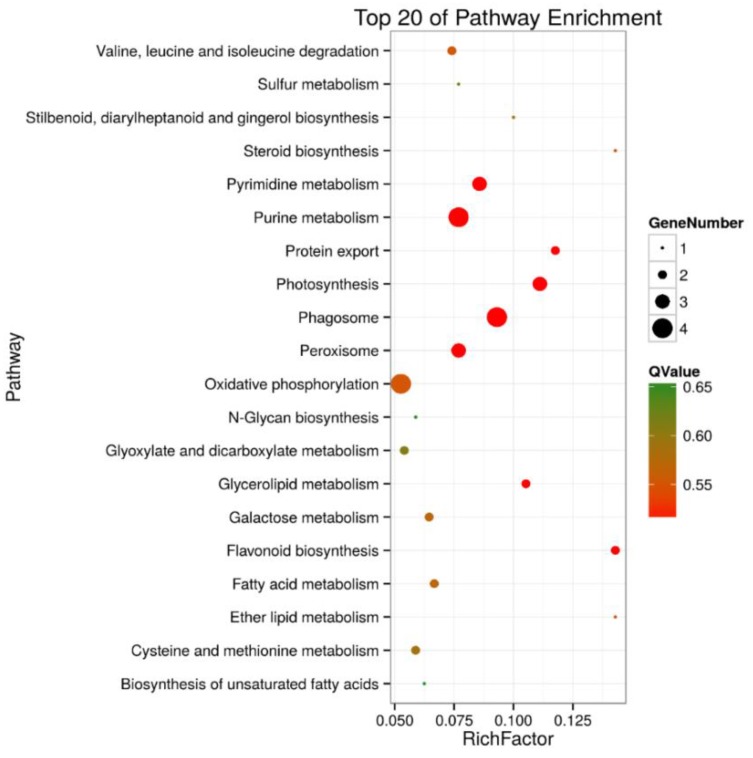
Top 20 pathway enrichments of DEPs in roots of salt-treated sugar beet plants compared with controlled plants. The *Y*-axis indicates the KEGG pathway, the *X*-axis indicates the rich factor. The dot size indicates the number of DEPs of the pathway, and the dot color indicates the *Q*-value. Rich factor indicates the number of differentially abundant proteins participating in a KEGG pathway as a proportion of proteins involved in the pathway in all identified proteins. A *P*-value less than 0.05 was defined as statistically significant.

**Figure 6:**
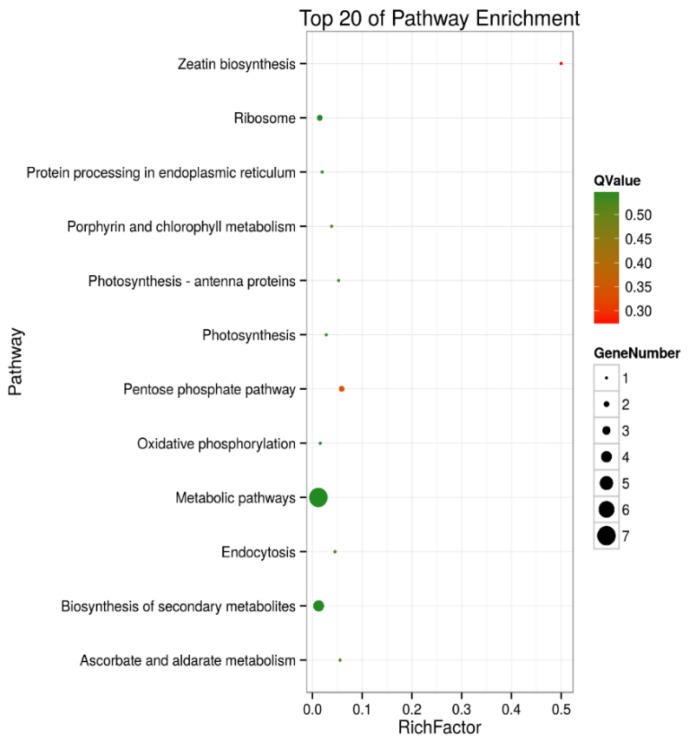
Top 20 pathway enrichments of DEPs in shoots of salt-treated sugar beet plants compared with controlled plants. The *Y*-axis indicates the KEGG pathway, the *X*-axis indicates the rich factor. The dot size indicates the number of DEPs of the pathway, and the dot color indicates the *Q*-value. Rich factor indicates the number of differentially abundant proteins participating in a KEGG pathway as a proportion of proteins involved in the pathway in all identified proteins. A *P*-value less than 0.05 was defined as statistically significant.

A KEGG pathway enrichment analysis of the 105 and 30 DEPs in roots and shoots was carried out in a Blast software (version 2.2.26), respectively. The results showed that in roots, 43 of the DEPs were annotated to 41 KEGG pathways, and the metabolic pathways (ko01100) were the primary pathway enrichment. The second was the biosynthesis of secondary metabolites (ko1110). These results indicated that metabolites are significantly changed during salt stress. It was observed that DEPs participated in the following tolerance related pathways, such as ribosome (ko03010, 6 DEPs), oxidative phosphorylation (ko00190, 4 DEPs), pyruvate metabolism (ko00230, 4 DEPs), phagosome (ko04145, 4 DEPs), peroxisome (ko04146, 3 DEPs), and glycerolipid metabolism (ko00561, 2 DEPs) (Table S3 and Figure 5). In shoots, 12 of the DEPs were annotated to 10 KEGG pathways, including the pentose phosphate pathway (ko00030, 2 DEPs), ascorbate and aldarate metabolism (ko00053, 1 DEP), oxidative phosphorylation (ko00190, 1 DEP), photosynthesis (ko00195, 1 DEP), photosynthesis- antenna proteins (ko00196, 1 DEP), porphyrin and chlorophyll metabolism (ko00860, 1 DEP), zeatin biosynthesis (ko00908, 1 DEP), ribosome (ko03010, 2 DEPs), protein processing in endoplasmic reticulum (ko04141, 1 DEP), and endocytosis (ko04144, 1 DEP) (Table S4, Figure 6).

In the KEGG pathway enrichment process, an R language was used for the hyper geometric algorithm. The functions of the DEPS were determined for the classification of pathways (Tables S3 and S4). In roots, the functions of the DEPs were mainly involved in metabolism, including carbohydrate, lipid, energy, nucleotide and amino acid metabolism as well as biosynthesis of other secondary metabolites. The second was genetic information processing, including translation, transcription, folding, sorting, and degradation. The third was cellular processing, including transport and catabolism, which occupied a lower proportion of the DEPs (Table S3). In shoots, the DEPs’ functions were also divided into three groups: metabolism, genetic information, and cellular processing. Metabolism included carbohydrate, energy, cofactors, and vitamins metabolism. Genetic information processing had translation, folding, sorting, and degradation. Cellular processing also mainly included transport and catabolism (Table S4).

### 2.5. qRT-PCR Analysis of Candidate Genes Encoding DEPs

**Figure 7:**
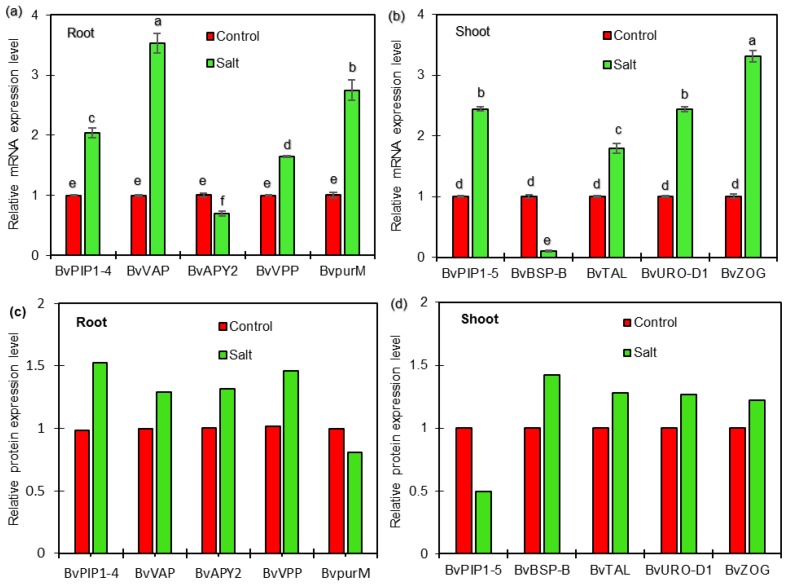
qRT-PCR expression analysis of selected genes with corresponding protein change in roots (**a**,**c**) and shoots (**b**,**d**) of sugar beet plants exposed control and 50 mM NaCl for 72 h. Relative mRNA levels in salt-treated plants were normalized against the control plants. Values are mean ± SE of three independent experiments. Columns with different letters indicate significant differences at *p* <0.05 (Duncan’s test). The relative protein expression levels of selected protein were derived from proteomics data of the present iTRAQ-based experiment. The selected proteins were BvPIP1-4 (Unigene0033986), BvVP (Unigene0011701), BvpurM (Unigene0024368), and BvVAP (Unigene0010790) in roots; BvPIP1-5 (Unigene0033990), BvTAL (Unigene0005386), BvURO-D1(Unigene0000482) and BvZOG (Unigene0016372) in shoots, respectively. *BvACTIN* was used as an internal control to normalize the transcript levels of all expression analyses.

To determine whether the DEPs are associated with transcriptional changes, qRT-PCR analysis was performed to detect the correlation between proteins and genes expression in roots and shoots of sugar beet seedlings exposed to salt treatment in the present study. The results showed that four genes, *BvPIP1-4* (Unigene0033986), *BvVP* (Unigene0011701), *BvpurM* (Unigene0024368), and *BvVAP* (Unigene0010790), were up-regulated, and one gene, *BvAPY2* (Unigene0014152), was down-regulated at transcriptional levels in roots (Figure 7a). It was observed that four genes, *BvPIP1-5* (Unigene0033990), *BvTAL* (Unigene0005386), *BvURO-D1* (Unigene0000482), and *BvZOG* (Unigene0016372), were up-regulated at transcriptional levels in shoots (Figure 7b). Further analysis indicated that six of the selected proteins, including BvPIP1-4, BvVP, and BvVAP in roots and BvTAL, BvURO-D1, and BvZOG in shoots, showed increased abundance, consistent with the changes in abundances of the corresponding proteins as revealed from the iTRAQ-based experiment (Tables S1 and S2, Figure 7c,d), indicating a good correlation between the expression levels of protein and mRNA and a high quality of the quantification results in the present study. Interesting, at the transcriptional level, *BvAPY2* and *BvBSP-B* were found to be down-regulated, and *BvpurM* and *BvPIP1-5* were up-regulated in salt-treated plants compared with control plants (Figure 7a,b). However, enhanced abundance of BvAPY2 and BvBSP-B and reduced abundance of BvpurM and BvPIP1-5 were detected in salt-responsive proteome in the present study. These results indicated that their protein levels might be determined not only at the transcriptional level but also at the post-translational level.

## 3. Discussion

To cope with salt stress, sugar beet plants have evolved complex salt-responsive signaling and metabolic processes at the molecular, cellular, organ, and whole-plant levels [15,17,19]. In our previous study, morphological and physiological changes in sugar beet seedlings were observed which represented the plant’s response to salt stress [23,24]. Yu et al. [18] reported that in sugar beet M14, a total of 2182 proteins were identified and 114 proteins showed differential levels under salt stress. The present study involved a comparative analysis of salt responses to roots and shoots of sugar beet ‘Gantang7’ seedlings using a quantitative proteomic approach. Among 3922 and 2510 identified proteins, respectively, a total of 105 proteins in roots and 30 proteins in shoots responded to NaCl. The functions of these salt-responsive proteins and their main pathways are discussed further below.

### 3.1. Proteins Involved in Carbohydrate and Energy Metabolism

Salt stress alters the abundance of many proteins involved in carbon and energy metabolism, including glycolysis, the tricarboxylic acid cycle (TCA), and the pentose phosphate pathway (PPP) in root and PPP and ascorbate and aldarate metabolism in shoots of sugar beet. It was found that the level of ATP-dependent 6-phosphofructokinase 5 (PFK5, Unigene0011604) was increased in roots of sugar beet under salt treatment. PFK5, a key regulatory enzyme in the glycolytic pathway, catalyzes the phosphorylation of D-fructose 6-phosphate to fructose 1,6-bisphosphate by ATP, the first committing step of glycolysis [25]. It was reported that PFKs gene family consists of seven members in *Arabidopsis thaliana* [26]. In our experiment, a phosphoglucomutase protein (Unigene0030344), which facilitates the interconversion of glucose 1-phosphate and glucose 6-phosphate, displayed a decrease in abundance after 72 h of 50 mM NaCl treatment. It was also observed that the level of phosphoglucomutase was decreased in salt sensitive cultivar ‘SA0604’ but increased in the salt tolerant one ‘TNG67’ in rice (*Oryza sativa*) after 30 min of 250 mM NaCl stress [27].

One protein related to TCA was identified in this study. Malate dehydrogenase (MDH, Unigene0018571) is an enzyme that reversibly catalyzes the oxidation of malate to oxaloacetate using the reduction of NAD^+^ to NADH in the TCA cycle. There is evidence that overexpression of MDH gene enhanced the synthesis of organic acids and conferred tolerance to aluminum in transgenic alfalfa (*Medicago sativa*) [28]. In this study, the level of MDH (mitochondrial) was reduced in roots of sugar beet plants under salt condition. This suggests that the TCA cycle might be inhibited in sugar beet roots after 72 h of salt treatment. A similar result was observed in upland cotton (*Gossypium hirsutum*) under salt stress [29]. However, the level of MDH (cytoplasmic) was increased in roots of cucumber (*Cucumis sativus*) and in young panicles of rice under salt stress [30,31]. A possible reason is that the MDH cellular localization was different in each species. It was well known that some ROS-scavenging systems need the PPP pathway that produces NADPH under salt stress conditions [29,32]. Transaldolases (TAL1, Unigene0005386; TAL2, Unigene0005387), the key enzymes of the non-oxidative phase of the PPP pathway [32], were enhanced in shoots of sugar beet plants exposed to salt.

Energy metabolism is often suppressed when plants are exposed to salt stress. Under salt stress, salt-tolerant plants can improve the synthesis of organic osmotic substances and increase osmotic pressure through energy metabolism improvement for stress adaptation [33]. Energy metabolism mainly included oxidative phosphorylation (OXPHOS) and photosynthesis in this study. OXPHOS is a fundamental and efficient ATP generating process for cellular respiration, consisting of four respiratory chain protein complexes (complexes I–IV), the ATP synthase complex (complex V), and two mobile electron transporters (ubiquinone and cytochrome c) [34,35]. In our study, four proteins related to OXPHOS were identified. Pyrophosphate-energized vacuolar membrane proton pump (V-H^+^-PPase, Unigene0011701), V-type proton ATPase subunit c1 (V-H^+^-ATPase, Unigene0000487), V-H^+^-ATPase 16 kDa proteolipid subunit (Unigene0010790), and NADH dehydrogenase subunit (Unigene0026083) were increased in roots of sugar beet after 72 h of 50 mM NaCl treatment. Similar results were found in previous studies of proteomics in *Carex rigescens* [35], *Halogeton glomeratus* [36], and *Brassica napus* [37]. V-H^+^-PPase and V-H^+^-ATPase are two proton transport systems in the plant vacuolar membrane; these systems create electrochemical potential gradients across the tonoplast and transport many metabolites [38,39]. These proton pumps have been identified in several proteomic studies and are also considered as important salt-responsive marker proteins [37,40,41,42]. Overexpression of V-H^+^-PPase or V-H^+^-ATPase resulted in enhanced salt tolerance in transgenic tobacco [43] or *Broussonetia papyrifera* [44]. These results indicated in the presence of salt that sugar beet is able to adapt by modulating proton transport and improving ATPase and PPase synthesis.

Photosynthesis is one of the most important processes to be affected by salt. The effects of salt stress on photosynthesis are either direct, such as diffusion limitations through the stomata and the mesophyll, and changes in photosynthesis metabolism, or indirect, such as the oxidative stress resulting from the superimposition of multiple stresses [45]. In this study, two proteins involved in photosynthesis were also identified in sugar beet shoots under salt treatment. The level of PsbQ-like protein 2 (Unigene0024040) was decreased. It was reported that PsbQ is a luminal extrinsic protein component that regulates the water splitting activity of photosystem II (PSII) in plants [46]. The down accumulation of chlorophyll a-b binding protein 1A (Unigene0012485) was also observed in shoots under salt condition. The decreased abundance of these proteins implied that photosynthesis was down-regulated in sugar beet exposed to salt treatment.

### 3.2. Liqid Metabolism and Secondary Metabolite Biosynthesis Retated Protein

In this study, two proteins related to fatty acid metabolism were identified in roots of sugar beet. It is clear that a protein 3-ketoacyl-CoA thiolase 2 (KAT2, Unigene0023974), was reduced, whereas another one, Acyl-CoA dehydrogenase (ACAD) family member 10 (Unigene0030654), was increased at protein levels under salt stress. It has been shown that KAT2 has a broad chain-length specificity for its substrates and is involved in degradative pathways such as fatty acid β-oxidation [47,48]. In *Arabidopsis*, the triacylglycerol (TAG) and the total amount of lipid decreases rapidly during growth of wild-type plants, but the amounts remain unchanged in *kat2* mutant, indicating that KAT2 is required for TAG catabolism, and that products of other KAT genes cannot substitute for it during *kat2* plant growth [47]. ACADs are a class of enzymes that function in catalyzing the initial step in each cycle of fatty acid β-oxidation in the mitochondria of cells [49]. In our study, the reduction of KAT2 might block fatty acid β-oxidation, whereas the increase of ACAD 10 might probably enhance these biochemical processes. However, the roles of these two proteins in salt tolerance of sugar beet plants still need to be addressed in future studies. In addition, our proteomics date showed that the level of obtusifoliol 14-α demethylase-like (Unigene0022787), which is related to steroid biosynthesis, was increased in sugar beet roots under salt treatment. The obtusifoliol 14-α demethylase (CYP51) is a member of the cytochrome P450 gene family, which catalyzes the oxidative removal of the 14-α methyl group of obtusifoliol in plants and some bacteria [50,51]. In these organisms, this enzymatic oxidative step is considered to be one of the key steps of sterol biosynthesis [50,52]. These results implied that the increase of CYP51 could probably enhance the sterol biosynthesis pathway to maintain cell membrane fluidity and permeability, thus improving salt tolerance in sugar beet.

Secondary metabolism plays an important role in osmotic adjustment and oxidation reactions under salt condition [35]. Herein, some peroxidases related to phenylpropanoid biosynthesis were arrested and down-regulated after salt stress. Previous studies showed that with the decrease of peroxidase, the synthesis of phenylpropanoids was repressed after salt stress [53]. However, our results showed that the protein trans-cinnamate 4-monooxygenase (C4H, Unigene0000590) only responded in roots with an obvious increase. Similar results are also reported for *Arabidopsis* [41]. However, the expression level of C4H was significantly higher in shoots than roots in *C. rigescens* [35]. The accumulation of C4H in roots might be important in sugar beet plants responding to salt stress.

Flavonoid biosynthesis is an integral part of secondary metabolism, so it should be considered within a cellular metabolism context [54]. We observed that the protein, naringenin,2-oxoglutarate 3-dioxygenase (F3H, Unigene0023544) reduced at protein level in sugar beet roots after 72 h of 50 mM NaCl. It is well known that F3H catalyzes the 3-β-hydroxylation of 2S-flavanones to 2R, 3R-dihydroflavonols which are intermediates in the biosynthesis of flavonols [55]. Therefore, F3H is thought to be a key enzyme in flavonoid biosynthesis. In this study, up-regulation of this protein could accelerate the flavonoid biosynthesis pathway in response to salt in sugar beet.

Zeatin is the most active and ubiquitous of the naturally occurring cytokinins [56]. The zeatin O-glucoside (ZOG), found in all plants examined, is considered to be important in cytokinin (CK) transport, storage, and protection against cytokinin oxidases [57]. In the present study, zeatin O-glucosyltransferase (Unigene0016372), a protein related to zeatin biosynthesis, was obviously increased in sugar beet exposed to salt. It was observed that zeatin O-glucosyltransferase OsZOG1 regulates root and shoot development and formation of agronomic traits in rice [58]. Overexpression of a zeatin O-glucosyltransferase gene significantly enhanced the accumulation of both CK and auxin in roots and lower leaves of transgenic tobacco plants when subjected to water deficit [59]. The increase of ZOG1 in shoots could result in the elevation of CK O-glucoside content to cope with environmental stress in sugar beet.

### 3.3. Transcription and Protein Synthesis Related Protein

It was well known that under salt condition, many induced proteins from a signaling network regulate transcription and translation of proteins as key processes for response and adaptation to salt stress [21]. In the spliceosome, two proteins related to transcription, formin-like protein 5 (Unigene0012710) and U2 small nuclear ribonucleo protein B (Unigene0028062), showed significant reduction of abundance in sugar beet roots under salt treatment in this study. This implied that transcription was affected under salt treatment. Protein turnover, the balance between synthesis and degradation, is one of the many forms of regulation that is employed to achieve a unified cellular response [29]. Several proteins, involved in translation, processing, and degradation of protein were identified in our study. It was found that the levels of 40S ribosomal protein S8 (Unigene0009721), 40S ribosomal protein S12 (Unigene0014183), and 60S ribosomal protein L5 (Unigene0007343) were decreased, whereas the abundance of 40S ribosomal protein SA (Unigene0020287) and 60S ribosomal protein L15 (Unigene0033493) were increased in roots of sugar beet under salt stress. It was also observed that the abundance of 60S acidic ribosomal protein P0 (Unigene0023109) was increased and 50S ribosomal protein L18 (Unigene0024177) was decreased in shoots of sugar beet in response to salt. Ribosomes have been shown to be essential ribonucleoprotein complexes that are engaged in translation and thus play an important role in metabolism, cell division, and growth [35,45]. The expression levels of some of the ribosomal proteins increased while some specific ribosomal components decreased under salt conditions as observed in *Arabidopsis* [41], alfalfa [45], upland cotton [29,60], and *C. rigescens* [35]. Similar results were also reported in the sugar beet M14 line [18,21]. The changes in protein synthesis investigated in our study further confirm the notion that salinity stress represses the synthesis of protein. However, the activities of specific protein synthesis may be enhanced if these proteins are of particular importance to salt tolerance. In addition, our data showed lower expression of elongation factor 1-α (Unigene0008589) under salinity conditions. However, this protein displayed higher abundance in upland cotton [29] and *Halogeton glomeratus* [36] when exposed to salt stress. The differential regulation of different components of the translation machinery implies that a complicated regulation mechanism may control the synthesis of protein to help plants cope with salt treatment. 

### 3.4. Protein Folding, Sorting and Degradation

Under salt stress, some proteins are misfolded or wrongly assembled, so plants express many proteins to assist refolding and sorting again or degrade these abnormal proteins [61]. In the present study, four proteins related to folding, sorting, and degradation were identified. The signal recognition particle (SRP) is a multimeric protein, which along with its conjugate receptor, is involved in targeting secretory proteins to the rough endoplasmic reticulum (RER) membrane in eukaryotes [62]. In our proteomics data, both subunit α (Unigene0003201) and subunit β (Unigene00151740) of signal recognition particle receptor (SRPR) increased at protein level in sugar beet roots under salt condition. Dolichyl-diphosphooligosaccharide-protein glycosyltransferase subunit STT3B is an essential subunit of the N-oligosaccharyltransferase (OST) complex and plays an important role in the ER-associated degradation (ERAD) pathway that mediates ubiquitin-dependent degradation of misfolded endoplasmic reticulum proteins by mediating N-glycosylation of unfolded proteins [63]. In this study, STT3B (Unigene0021730) was induced at protein level in sugar beet roots after 72 h of 50 mM NaCl treatment. Consistent with this observation, the level of STT3B was also significantly increased in the sugar beet M14 line when subjected to salt stress [21]. Our data also showed that transaldolase 1 (Unigene0005386), which is involved in protein processing in ER, increased the expression abundance at both protein and transcriptional levels. All these proteins function to maintain normal protein folding, as well as repair and renaturation of stress-damaged proteins.

### 3.5. Protein Involved in Transport

In general, salt stress causes ionic imbalance and toxicity, and plants have to re-establish cellular ion homeostasis by regulating cross-membrane transport [18]. A total of two V-type proton ATPase was increased in sugar beet roots after 72 h of salt treatment in our study. H^+^-ATPase plays an important role in the maintenance of ion homeostasis in plant cells [39,40]. Overexpression of V-H^+^-ATPase conferred tolerance to environmental stresses (salt, saline–alkali, and osmotic stress) in transgenic alfalfa [64] and *Arabidopsis* [65]. Further analysis indicated that under high salinity transgenic plants compartmentalized more Na^+^ and showed the enhanced osmotic adjustment in the leaves compared with wild-type plants [65]. Therefore, the increased activities of these enzymes may be an effective strategy for osmotic adjustment, which enhances the Na^+^ compartmentalization into vacuoles and reduces Na^+^ accumulation in the cytosol of sugar beet plants.

Microtubules function in a network for numerous cellular processes such as salt response [66]. Tubulin is the major constituent of microtubules. This protein is related to plant salt-stress adaptation, constructing a microtubule skeletal structure in eukaryotic cells, controlling cell expansion and cell shape, and binding with GTP to participate in post-translational modification [67,68]. Our proteomics data showed that the abundance of tubulin α-2 chain (Unigene0007087) was increased while the level of tubulin β-1 chain (Unigene0008997) was decreased in roots of sugar beet in response to salt treatment. Salt-induced tubulin α chain and salt-reduced β-2/β-3 was previously identified indicating dynamic reorganization of microtubules [41,66].

Aquaporins (AQPs), which are also called water channels, belong to a highly conserved, major intrinsic protein family and interact with the cell membrane system to transport water and a variety of low-molecular-weight solutes [69,70]. PIPs and TIPs, two subfamilies of AQPs, are most abundant in plasma membrane and vacuolar membrane, respectively [71]. The level of ThPIP2;5 was increased by salt stress in *Tamarix hispida* seedlings, and its overexpression enhanced salt tolerance in transgenic *Tamarix* and *Arabidopsis* [72]. However, overexpression of barley (*Hordeum vulgare*) *HvPIP2;1* gene raised salt sensitivity in transgenic rice plants under salt stress [73]. The overexpression of AQPs in this plant may increase membrane water permeability and thus decrease cellular water conservation during periods of salt stress. Consistent with this observation, the expression levels of ZmPIP1 and ZmPIP2 in maize were also decreased under salt stress [74]. Thus, the regulation mechanism of AQPs under salt stress conditions is complicated and requires further study. Several AQPs proteins identified in the present study displayed different responses to salt treatment. Three PIPs (PIP1-4, Unigene0033986; PIP2-1, Unigene0020288; PIP2-1-like, Unigene0029552) and one TIPs (TIP1-3, Unigene0020288) showed higher abundance in roots, whereas one PIPs (PIP1-5, Unigene0033990) displayed lower abundance in shoots of sugar beet exposed to 50 mM NaCl. These proteins may play different roles in sugar beet plants adapting to salt stress. 

In conclusion, DEPs of 105 and 30 were identified in roots and shoots of sugar beet by iTRAQ-based proteomics technology, respectively. There are 46 proteins up-regulated and 59 proteins down-regulated in roots. The 13 up-regulated proteins and 17 down-regulated proteins were found in shoots, respectively. These DEPs were mainly involved in carbohydrate metabolism, energy metabolism, lipid metabolism, nucleotide and amino acid metabolism, biosynthesis of secondary metabolites, transcription, translation, protein folding, sorting, and degradation as well as transport. It is worth emphasizing that some novel salt-responsive proteins were identified, such as PFK5, PsbQ, MDH, KAT2, ACAD10, CYP51, F3H, SRPR, ZOG, V-H^+^-ATPase, V- H^+^-PPase, PIPs, TIPs, and tubulin α-2/β-1 chain. qRT-PCR analysis indicated that six of the selected proteins, including BvPIP1-4, BvVP, and BvVAP in roots and BvTAL, BvURO-D1 and BvZOG in shoots, showed good correlation between the expression levels of protein and mRNA. These novel proteins provide a good starting point for further research into their functions using genetic or other approaches. These findings significantly improve the understanding of the molecular mechanisms involved in salt tolerance of plants.

## 4. Materials and Methods

### 4.1. Plant Materials, Growth Conditions and Treatments

Seeds of sugar beet (*B. vulgaris* L. cv. ‘Gantang7’, a salt- and drought-tolerant cultivar) were provided by Wuwei Sannong Seed Technology Co., Ltd., Gansu province, China in middle August, 2014. Seeds were surface sterilized for 1 min in 75% ethanol (v/v) and rinsed 3 times with distilled water, soaked in distilled water for 24 h and then germinated at 25 °C in the dark for 72 h. Uniform seedlings were transplanted into plastic containers (5 × 5 × 5 cm; two seedlings/container) containing vermiculite and cultured with a modified Hoagland nutrient solution [22]. The seedlings were grown in a growth chamber, where the temperature was regulated at 26 °C in the day and 21 °C at night and the relative humidity averaged 75% and 65% for night and day, respectively. The light period was 16 h·d^−1^ and light intensity during the light period was between 500 and 600 μmol·m^−2^·s^−1^. In our previous study, NaCl concentration of 50 mM can stimulate the growth of plants and induce the expression of genes in sugar beet [24]. Therefore, in the present study, three-week-old plants were treated with modified Hoagland solution supplemented with 0 and 50 mM NaCl for 72 h, NaCl-free nutrient solution was used as a control. Treatment solutions were renewed every day. To ensure the ability to conduct statistical analyses, two biological replicates (12 plants per replicate) were used for the iTRAQ-based quantitative proteomic analysis and three biological replicates (12 plants per replicate) were used for qRT-PCR analysis. All the plants were arranged randomly in the same chamber, and their positions were rearranged each day.

At the end of the treatment, the plants were slightly washed with distilled water to remove surface salts and vermiculite. Roots and shoots (all of leaf blades and leaf petioles) of whole plants were separated and blotted; collected samples were wrapped in aluminum foil and flash frozen in liquid nitrogen and then stored at −80 °C until further use.

### 4.2. Protein Preparation

One gram of frozen mixed samples (roots or shoots) from 12 plants per replicate was ground into a fine powder using liquid nitrogen. The dry powder was dissolved with 200 µL lysis buffer (7 M Urea, 2 M thiourea, 4% CHAPS, 40 mM Tris-HCl, pH 8.5) containing 1 mM PMSF and 2 mM EDTA (final concentration). The samples were placed on ice for 10 min, and then 10 mM DTT was added to the samples. The samples were sonicated at 200 W for 5 min, and centrifuged at 15,000× *g* for 15 min at 4 °C. The sediment was discarded and the supernatant was transferred into a new tube, then 4× volumes of cold acetone were added containing 10% (v/v) TCA and 10 mM DTT, and incubated at −20 °C overnight. The sediment was collected by centrifugation at 13,000× *g* for 20 min. The sediment was re-suspended by adding 800 µL cold acetone (containing a final concentration of 10 mM DTT), and washed three times. The sediment was centrifuged at 13,000× *g* for 20 min, collected and then air-dried. Then 100 µL lysis buffer (7 M Urea, 2 M thiourea, 4% CHAPS, 40 mM Tris-HCl, pH 8.5) containing 1 mM PMSF and 2 mM EDTA was added to dissolve protein, then after 5 min 10 mM DTT was added. The samples were sonicated at 200 W for 5 min, and then centrifuged at 25,000× *g* for 15 min at 4 °C. The sediment was discarded and the supernatant was transferred into a new tube, then incubated at 56 °C for 1 h to eliminate disulfide bonds between proteins. After this, 55 mM IAM (SIGMA-ALDRICH, Burlington, MA, USA) was added to inhibit cysteine and incubated in the dark 1 h. The 4× volumes of cold acetone were added into the supernatant to precipitate protein species at −20 ° C over 2 h. The mixture was centrifuged at 3000× *g* for 5 min at 4 ° C, and the sediment was collected and air-dried for 15 min. The sediment was dissolved in 500 µL of 0.5 M TEAB (Applied Biosystems, Milan, Italy), and sonicated at 200 W for 15 min. Finally, the mixture was centrifuged at 3000× *g* for 15 min at 4 °C. The supernatant was transferred into a new tube, and the protein concentration was quantified using the Bio-Rad Protein Assay Kit (Bio-rad, Hercules, CA, USA) based on the Bradford method using BSA as a standard [75]. The protein in the supernatant was kept at −80 °C until further analysis.

### 4.3. iTRAQ Labeling and SCX Fractionation

An amount of 100 µg protein of each sample was transferred to a new tube and adjusted to a volume of 100 µL with 8 M urea, and 11 µL of 1 M DTT added and incubated at 37 °C for 1 h. Then the sample was added to a 10 K ultrafiltration tube (Millipore Co., Billerica, MA, USA), and centrifuged at 14,000× *g* for 10 min, 120 µL of 55 mM iodoacetamide was added, and the mixture was incubated at room temperature for 20 min in the dark, and the iodoacetamide removed by centrifugation. After substituting the Urea system three times with 100 mM TEAB in an ultrafiltration tube, the solution was digested with 1:50 Trypsin Gold (Promega, Madison, WI, USA) overnight. Following the trypsin digestion, the sample was collected by centrifugation over 12 min, and vacuum freeze-dried. Peptides were reconstituted with 50 µL of 500 mM TEAB solution. and then the iTRAQ labeling reagent (iTRAQ Reagents-8Plex, SCIEX) added to 50 µg enzymatically decomposed protein according to the manufacturer’s introductions. Each unit of iTRAQ reagent was thawed and reconstituted with 24 µL of isopropanol. The labels of roots and shoots samples were performed separately. Proteins in the shoots of the control plants and salt-treated plants were expressed as CK1-1/CK1-2 and T1-1/T1-2, respectively. The shoots samples were labeled with iTRAQ tags 117 and 118 (CK1-1/CK1-2), 119 and 121 (T1-1/T1-2). Proteins in roots of the control and salt-treated plants were expressed as CK2-1/CK2-2 and T2-1/T2-2, respectively. The roots samples were labeled with iTRAQ tags 119 and121 (CK2-1/CK2-2), 117 and 118 (T2-1/T2-2). The mixed solutions were cultured at room temperature for 2 h, then 100 µL ultrapure water was added to terminate the reaction over 15 min. The proteins of each channel were mixed and dried for use in a vacuum concentrator.

The samples of roots and shoots were redissolved in 100 µL of buffer A (20 mM ammonium formate aqueous solution, ammonia water adjusted to pH 10.0), and then separately mixed and put into an Ultremex SCX (strong cation exchange) column (4.6 × 250 mm, Waters Corporation, Milford, MA, USA) containing 5 µm particles, with the column connected to the Ultimate 3000 system (Thermo Fisher Scientific, Waltham, MA, USA). The separation was performed using a linear gradient of 5% to 45% buffer B (buffer B: 20 mM ammonium formate was added to 80% CAN and the ammonia adjusted to pH 10.0) for 40 min. The column was equilibrated for 15 min under initial conditions, the column flow rate was maintained at 1 mL·min^−1^, and the column temperature was maintained at 30 °C. A fraction was collected every 100 s, and a total of 24 fractions collected. The eluted peptides were pooled into 12 fractions according to the time of the collection. Each fraction was dried for use in a vacuum concentrator.

### 4.4. LC–ESI–MS/MS Analysis

Each fraction was added to 30 µL of buffer C (0.1% aqueous formic acid) to prepare a suspension, which was separated by nano-LC and analyzed by in-line electrospray tandem mass spectrometry. The experiment was performed on an Easy-nLC 1000 system (Thermo Fisher Scientific, Waltham, MA, USA), which was connected to an Orbitrap Fusion Tribrid mass spectrometer (Thermo Fisher Scientific, Waltham, MA, USA) equipped with an online nano electrospray ion source. The column (the trap column and the analytical column were connected in series, and the buffer phase used to equilibrate the column to allow the peptide in the sample to bind to the column) was equilibrated with buffer C at a flow rate of 300 µL·min^−1^ for 10 min. An amount of 10 µL of the polypeptide sample was loaded onto a capture column (Thermo Fisher Scientific Acclaim PepMap C18, 100 µm × 2 cm) at a flow rate of 10 µL·min^−1^, and followed by a linear gradient in the trap column and the analytical column (Acclaim PepMap C18, 75 µm × 15 cm): 2% to 40% buffer D (0.1% formic acid acetonitrile solution).

### 4.5. Protein Identification

Raw data files acquired from the mass spectrum of Orbitrap Fusion were converted to MGF format using mascot distiller. Protein information was identified using the Mascot software (Matrix Science, London, UK; version 2.3.02) against the GDPR0916_20151012.mascot_db.fa (26175 sequences) database. The type of search was MS/MS Ion search (MIS). Trypsin was specified as the digesting enzyme; Fragment mass tolerance was +/− 0.05 Da and peptide tolerance mass was 20 ppm; Oxidation (M) and Gln→pyro-Glu (N-term Q) were variable modifications, while carbamidomethyl (C), iTRAQ8plex (N-term), and iTRAQ8plex (K) were fixed modifications. In the identification of proteins, all spectra were involved in the identification, as long as the spectra-matched peptide could be identified as a protein and the protein was considered to be present. Only proteins with shared peptides were detected, and mascot software divided the identified proteins into a group.

For the same batch of samples, only the proteins of the peptides detected in some samples were not involved in the quantitative analysis. Only the proteins identified in all samples and unique spectra ≥2 were used to calculate the expression. It was used as reference with the peptide signal value of the control sample of roots or shoots used as a reference. The ratio of the signal value of the peptide in the salt-treated samples to the reference is considered as the relative expression of the peptide in the corresponding sample. The median of the relative expression levels of all peptides in each sample was normalized. When a protein had multiple unique peptides, the median relative expression level of all the unique peptide homogenizations of the protein was taken as the relative expression level of the protein.

According to the protein abundance level, when the difference multiple was >1.2 or <0.8 times and the *p* < 0.05 was statistically tested, it was regarded as a differentially expressed protein.

### 4.6. Bioinformatics Analysis

Functional annotation analysis of all identified proteins was performed by Gene Ontology (GO) database (http://www.geneontology.org) (accessed on 25 May 2000. The Cluster of Orthologous Groups (COG) of proteins database (http://www.ncbi.nlm.nih.gov/COG/) (accessed on 24 October 1997 was used to predict the possible functions of these proteins and perform function classification statistics. Pathways enrichment analysis of differentially expressed proteins was performed using the Kyoto Gene and Genomic Encyclopedia (KEGG) database (http://www.genome.jp/kegg/) (accessed on 1 January 1999).

### 4.7. Quantitative Reverse Transcription PCR (qRT-PCR)

Total RNA was extracted using the Trizol method, and the synthesis of the first strand of cDNA was carried out using a reverse transcription kit (Takara, Shiga, Japan), and the gene expression levels of the selected protein were detected by a Real-time PCR Kit (Takara). The primers were designed using primer premier 5.0 software, the sequences are listed in Table S5. Among them, *BvACTIN* was used as an internal control to normalize the transcript levels of all expression analyses. The expression levels were determined with the 2^−ΔΔCT^method [76].

### 4.8. Statistical Analysis

Data for qRT-PCR were performed by one-way analysis of variance (ANOVA) using statistical software (SPSS 19.0, Chicago, IL, USA). Duncan’s multiple range tests were used to detect significant difference between means at a significant level of *p* < 0.05.

## Supplementary Materials

Supplementary materials can be found at http://www.mdpi.com/1422-0067/19/12/3866/s1. Figure S1. Basic information statistics of protein in roots and shoots of sugar beet. Figure S2. Distribution of protein mass distribution in roots and shoots of sugar beet. Figure S3. Distribution of peptide length in roots and shoots of sugar beet. Figure S4. Distribution of the proteins’ sequence coverage in roots (a) and shoots (b) of sugar beet. Figure S5. Distribution of the number of peptides in roots and shoots of sugar beet. Table S1. DEPs annotation in roots of sugar beet. Table S2. DEPs annotation in shoots of sugar beet. Table S3. KEGG analysis of DEPs in roots. Table S4. KEGG analysis of DEPs in shoots. Table S5. Primers used in qRT-PCR.

## Abbreviations

2-DE	Two-dimensional gel electrophoresis
ACAD10	Acyl-CoA dehydrogenase family member 10
AQPs	Aquaporins
BSP-B	Bark storage protein B-like
C4H	Trans-cinnamate 4-monooxygenase
CK	Cytokinin
COG	Cluster of Orthologous Groups
CYP51	Obtusifoliol 14-α demethylase
DEPs	Differentially expressed proteins
ERAD	ER-associated degradation
F3H	Naringenin,2-oxoglutarate 3-dioxygenase
GO	Gene Ontology
iTRAQ	isobaric Tags for Relative and Absolute Quantitation
KAT2	3-ketoacyl-CoA thiolase 2
KEGG	Kyoto Encyclopedia of Genes and Genomes
MDH	Malate dehydrogenase
MS	Mass spectrometry
OST	N-oligosaccharyltransferase
OXPHOS	Oxidative phosphorylation
PFK5	ATP-dependent 6-phosphofructokinase 5
PIPs	Plasma membrane intrinsic proteins
PPP	Pentose phosphate pathway
PSII	Photosystem II
purM	Phosphoribosylformylglycinamidine cyclo-ligase
qRT-PCR	quantitative Reverse Transcription-Polymerase Chain Reaction
RER	Rough endoplasmic reticulum
SCX	Strong cation exchange
SRP	Signal recognition particle
SRPR	Signal recognition particle receptor
TAG	Triacylglycerol
TAL	Transaldolases
TCA	Tricarboxylic acid cycle
TIPs	Tonoplast intrinsic proteins
URO-D1	Uroporphyrinogen decarboxylase 1
VAP	V-type proton ATPase subunit c1
V-H+-ATPase	V-type proton ATPase
V-H+-PPase	Pyrophosphate-energized vacuolar membrane proton pump
VP	Pyrophosphate-energized vacuolar membrane proton pump
ZOG	Zeatin O-glucoside
