# Supplementary material for: iTRAQ-Based Comparative Proteomic Analysis Provides Insights into Molecular Mechanisms of Salt Tolerance in Sugar Beet (Beta vulgaris L.)
Source: Int J Mol Sci. 2018 Dec 4;19(12):3866. doi: 10.3390/ijms19123866 (PMC6321137; doi:10.3390/ijms19123866)
Supplement: Supplementary file 1 [file ijms-19-03866-s001.zip › Supplementary Materials R2/Figures S1-S5.docx]

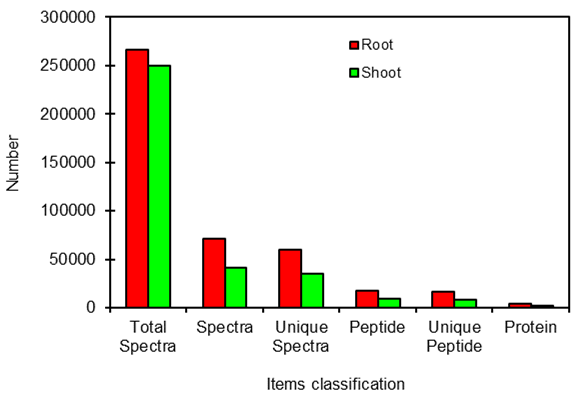


**Figure S1.** Basic information statistics of protein in root and shoot of sugar beet. Unique spectra: Spectra that matched unique peptides. Unique peptide: A protein-specific peptide.


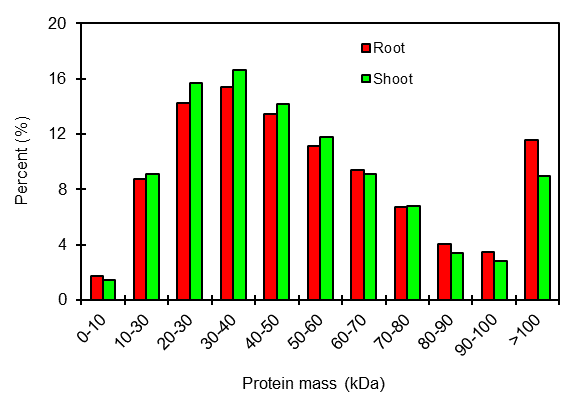


**Figure S2.** Distribution of protein mass distribution in root and shoot of sugar beet. The *X* axis represents the molecular weights (kDa) of the identified proteins, and the *Y* axis represents the number of proteins.


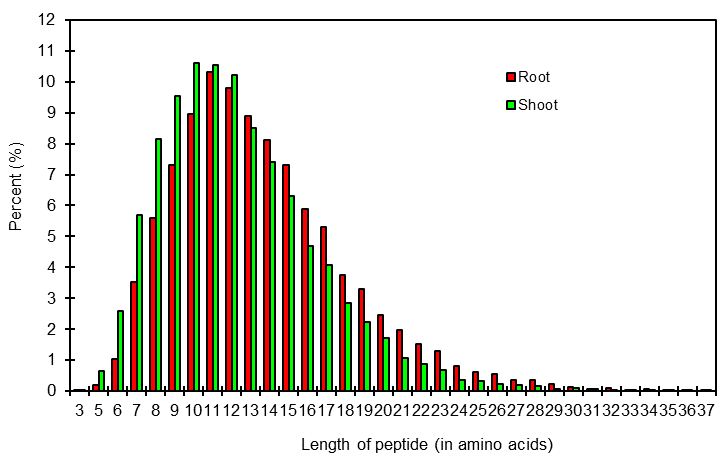


**Figure S3.** Distribution of peptide length in root and shoot of sugar beet. The *X* axis represents the length of the peptides, and the *Y* axis represents the number of proteins.


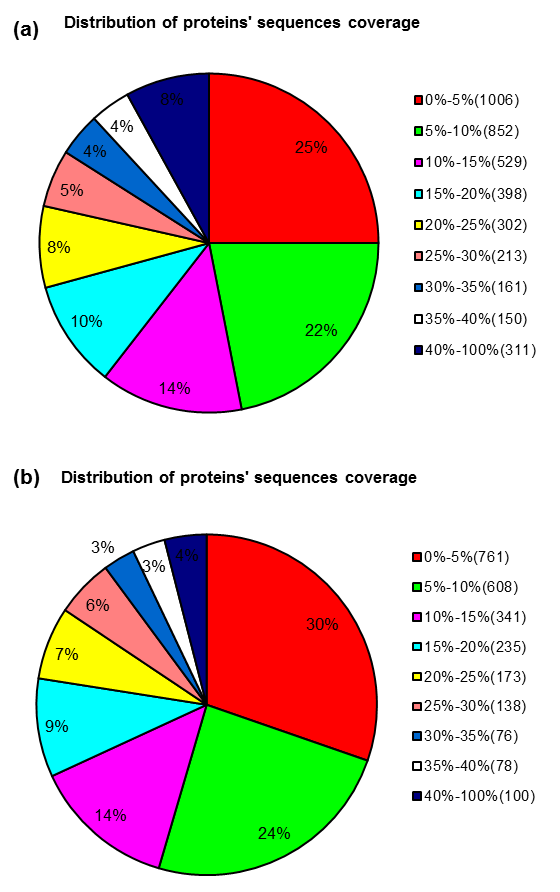


**Figure S4.** Distribution of the proteins’ sequence coverage in root (a) and shoot (b) of sugar beet. The different colors represent the coverage range of the different sequences, and the pie chart displays the proportion of the number of the different proteins within the scope of coverage in the total protein amount.


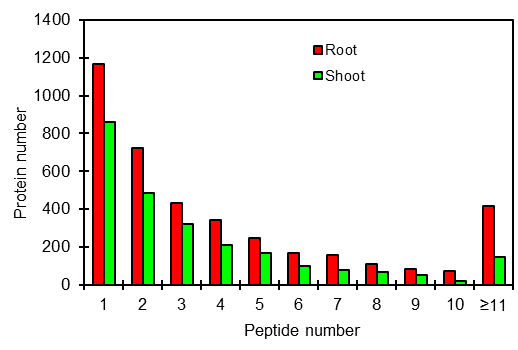


**Figure S5.** Distribution of the number of peptides in root and shoot of sugar beet. The *X* axis represents the scope of the number of identified peptides, and the *Y* axis represents the number of proteins.
